# Mycobacterium gordonae Infection in a Patient With Non-small Cell Lung Cancer

**DOI:** 10.7759/cureus.54689

**Published:** 2024-02-22

**Authors:** Alissa I Elanjian, Jason P Law, Borys Hrinczenko

**Affiliations:** 1 Medical Education, Michigan State University College of Human Medicine, East Lansing, USA; 2 Internal Medicine, Michigan State University College of Human Medicine, East Lansing, USA; 3 Oncology, Michigan State University College of Human Medicine, East Lansing, USA

**Keywords:** advanced non-small cell lung cancer, pathogenicity, pulmonary oncology, clinical infectious disease, nontuberculous mycobacteria (ntm), mycobacterium gordonae

## Abstract

*Mycobacterium gordonae* (MG) is one of the least pathogenic nontuberculous mycobacteria (NTM). We report an unusual case of MG infection in a patient with newly diagnosed lung cancer. A 61-year-old woman presented with shortness of breath and weight loss. Six months prior to admission, she was diagnosed with MG infection based on positive sputum cultures and bronchioalveolar lavage. Despite anti-mycobacterial therapy, her symptoms worsened and she lost approximately 100 pounds. A transbronchial biopsy obtained one week prior to admission revealed adenocarcinoma of the lung. At admission, vital signs were normal, and a physical exam revealed bilateral crackles. Computed tomography (CT) scan of the chest revealed infiltrates with ground-glass opacity. The patient was admitted to the oncology service for evaluation. Our findings suggest that symptomatic individuals with positive cultures of MG should proceed with extensive workup for possible underlying lung cancer especially if not responding to anti-mycobacterial therapy.

## Introduction

Atypical or nontuberculous mycobacteria (NTM) are generally free-living organisms that are ubiquitous in the environment. Advanced laboratory techniques can now isolate and identify NTM. The frequency of isolating them has recently increased. Their relevance to human disease has become more apparent as the incidence of tuberculosis declined. Currently, the most common nontuberculous species causing human disease in the United States are the slowly growing species *Mycobacterium avium* complex (MAC) and the rapidly growing species *M. abscessus*. Another slowly growing species, *M. kansasii*, has the greatest virulency. Less common human pathogens include the slowly growing species *M. marinum*, *M. xenopi*, *M. simiae*, *M. malmoense*, and *M. ulcerans*, as well as the rapidly growing species *M. fortuitum* and *M. chelonae* [1]. Certain relatively common laboratory isolates such as *Mycobacterium gordonae *(MG) are important to clinicians because they often are contaminants and commonly are not true pathogens. MGis one of the least pathogenic, slowly growing *Mycobacterium* species. In this discussion, we report an unusual case of MG infection in a patient with newly diagnosed non-small cell lung cancer.

## Case presentation

A 61-year-old woman presented at the oncology clinic with a long history of shortness of breath due to emphysema. The patient has a significant history of tobacco use, totaling 60 pack-years; however, she successfully quit smoking 20 years ago. Additionally, her family history includes her mother passing away at age 64 due to uterine cancer and her sister passing away at age 24 due to complications from pancreatitis. She mentioned that she had always been told she had pneumonia. A chest computed tomography (CT) scan done nine months prior revealed a 1.5 cm pre-tracheal lymph node, severe bilateral emphysema, and consolidation changes in the right infrahilar region and medial right lung base measuring 4.5 x 3.8 cm, along with multiple ground-glass opacities and confluent consolidation in the left lung base. 

A month after the CT scan, she was hospitalized for a cough and weight loss and was diagnosed with an MG pulmonary infection. She believed she had acquired the infection from an old showerhead. The patient started on anti-tuberculosis medications (rifampin, ethambutol, and ciprofloxacin). During the oncology clinic evaluation, she reported symptoms including shortness of breath, bone pain, headache, palpitations, restless legs at night, anorexia, and a weight loss of 100 lbs. She was receiving oxygen therapy at 3 LPM via nasal cannula. 

**Figure 1:**
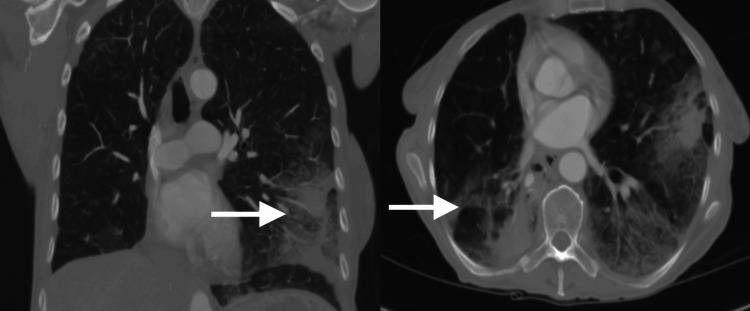
Coronal (left) and axial (right) chest CT views of the infected patient. White arrows depict diffuse bilateral infiltrates with ground-glass opacity and bibasilar pleural effusions. CT: computed tomography

A week before her clinic evaluation, she underwent a bronchoscopy with biopsies, which revealed adenocarcinoma with a bronchioalveolar pattern. She denied fevers or hemoptysis, and her Eastern Cooperative Oncology Group (ECOG) performance status was 1. On physical examination, she appeared cachectic, with clear lung sounds and regular heart rate and rhythm without murmurs. Laboratory tests showed a normal white blood cell count of 5.7 x 10^9^/L, mild anemia with a hemoglobin of 11.6 g/dL, and a normal platelet count of 237 x 10^9^/L. However, her serum calcium level was elevated at 13.1 mg/dL, creatinine was elevated at 2.01 mg/dL, blood urea nitrogen (BUN) was elevated at 43 mg/dL, and glomerular filtration rate (GFR) was low and calculated at 25 mL/min/1.73 m^2^. Chest X-ray showed extensive opacification of the left chest with bibasilar effusions. CT scan of the chest revealed diffuse bilateral infiltrates with ground-glass opacity and bibasilar pleural effusions (Figure 1).

She was admitted to the hospital for hypercalcemia and renal insufficiency, where she was treated with saline diuresis and zoledronic acid, leading to the resolution of her hypercalcemia and renal insufficiency. Subsequently, she was diagnosed with a stage IV adenocarcinoma of the lung and underwent treatment with four cycles of carboplatin and pemetrexed with an excellent response.

## Discussion

MGis a nontuberculous scotochromogenic mycobacteria named after Ruth E. Gordon, the American bacteriologist who discovered it. MG is also referred to as the "tap water bacillus" because it is frequently isolated in tap water and old showerheads and was previously known as *Mycobacterium aquae*. The prevalence of MG isolation in sputum is increasing. A study examining the growth of nontuberculous cases in Poland reported that the number of clinical samples containing MG rose from 66 cases in 2013 to 96 cases in 2017 [2].

The association of *Mycobacterium* tuberculosis and lung cancer has been well described in the medical literature. In a cohort study, Yu and colleagues [3] found that patients were 11 times more likely to develop lung cancer with tuberculosis than patients without tuberculosis, yielding an adjusted hazard ratio of 3.32 (95% CI: 2.70, 4.09). A systematic review by Liang and colleagues further concluded that an association exists between prior tuberculosis and lung cancer (95% CI: 1.93, 6.11). Cancer patients have also been identified as a high-risk group for developing tuberculosis (95% CI: 1.42, 1.96) [3]. Despite these findings, there is no clear causal mechanism for the link between tuberculosis and lung cancer. 

The clinical symptoms and radiological findings of NTM lung infection can mimic cancer or pose a diagnostic dilemma in a patient with cancer. Several reported cases from the literature described the occurrence of lung cancer in patients with NTM lung infection. In one case series, eight patients with infection had either concurrent or subsequent lung cancer. The most common cancer type was adenocarcinoma found in six of the patients followed by squamous cell carcinoma [4]. Multiple case reports described the occurrence of lung cancer in a previously infected lung with NTM (Table 1).

**Table 1:** Summary of case reports of lung cancer occurrence in patients with nontuberculous mycobacterial infection.

Class of *Mycobacterium*		Type of *Mycobacterium*	Type of lung cancer	Case reported
Slowly growing	Nonchromogens	*M. avium* complex	Adenocarcinoma	Taira et al. [5]
		M. malmoense	Non-small cell	Lapierre et al. [6]
		M. xenopi	Squamous cell	Doshi et al. [7]
		M. intracellulare	Undifferentiated metastatic	Garg et al [8]
	Photochromogens	M. kansasii	Large cell	Domej et al. [9]
Rapidly growing		M. fortuitum	Adenocarcinoma	Zhang and Chen [10]

MG infection is a rare disease. In a review of seven patients with sputum culture-positive MG, Chang et al. [11] found that the colonization of MG is more common in patients with either local or general immunosuppression. Infection with this organism may be a surrogate marker of an immunosuppressive disorder such as cancer. 

Our patient had a history of shortness of breath and weight loss with MG isolates found in her sputum. According to the American Thoracic Society (ATS) and Infectious Diseases Society of America (IDSA), the criteria for diagnosing nontuberculous mycobacterial pulmonary disease requires three components. Clinically, pulmonary or systemic symptoms must exist. Radiologic examinations must also be performed, indicating cavitary or nodular opacities from a chest radiograph or bronchiectasis and small nodules from a CT scan. Finally, any of the following microbiologic scenarios must be identified: positive NTM cultures from at least two sputum samples, a positive culture from at least one bronchial lavage or wash, a lung biopsy with histological features (acid-fast bacteria or granulomas) along with a positive culture for NTM, or a biopsy showing NTM features with at least one positive culture from sputum or bronchial washing. Our patient fit this criteria for pathogenic infection by MG in that there were 1) repeated isolation of MG, 2) isolation of the organism in the absence of other mycobacteria, and 3) clinical features in keeping with mycobacterial infection. Despite similarities between the case and MG infection protocols, the ATS and IDSA guidelines clarify that MG, in particular, seldom induces disease states in humans. Because of its limited pathogenicity, diagnostic procedures for MG should include more extensive testing than the general NTM guidelines. ATS and IDSA recommend strong symptomatic and radiologic findings, in addition to obtaining multiple positive cultures over a greater period of time [12].

Whether NTM infection is associated with lung cancer similar to *Mycobacterium* tuberculosis is debatable. Comparable to tuberculosis and also evident in NTM case reports (Table 1), prior MG lung infection may be linked with lung cancer. The inflammatory response from MG infection is a possible risk for the development of lung cancer since cancer developed in the same infected lung. However, MG is a common NTM isolate in sputum. Most isolates of this organism do not represent true infection. In our patient, clinical symptoms of MG did not improve with anti-mycobacterial therapy. The patient continued to be treated with antibiotics for six months despite a lack of response, which led to a late diagnosis of non-small cell lung cancer.

Greater research is necessary to explore links between MG and lung neoplasms since the association between NTM lung infections and cancer is still unclear. Our case report also calls for increased provider education on NTM and the diagnostic dilemma posed by infection and possible concurrent underlying malignancy. This awareness is crucial for the early detection of diseases, leading to timely treatment interventions and better prognoses.

## Conclusions

This article describes the experience of a patient who received an MG diagnosis based on positive sputum cultures and bronchioalveolar lavage, but did not respond to anti-mycobacterial therapy and was eventually diagnosed with lung adenocarcinoma. The case report and current NTM diagnostic criteria speak to the necessity of greater research on NTMs like MGand their potential to induce or exacerbate lung malignancies, as well as enhanced provider familiarity with the guidelines for treating NTMs of variable pathogenicity. Ultimately, the presence of NTM isolates in the sputum of a symptomatic patient should warrant a more extensive workup for a possible immunosuppressive disorder, especially if the patient is not responding to anti-mycobacterial therapy. Failure to consider alternative differentials in individuals infected by MG may result in a delayed diagnosis and less favorable prognosis of a concurrent immunosuppressive disorder such as cancer.
